# Cannabis and Paternal Epigenetic Inheritance

**DOI:** 10.3390/ijerph20095663

**Published:** 2023-04-27

**Authors:** Filomena Mazzeo, Rosaria Meccariello

**Affiliations:** 1 Dipartimento di Scienze Economiche, Giuridiche, Informatiche e Motorie, Università di Napoli Parthenope, Nola, 80035 Naples, Italy; 2 Department of Economics, Law, Cybersecurity and Sports Sciences, University of Naples “Parthenope”, Nola, 80133 Naples, Italy; 3 Dipartimento di Scienze Motorie e del Benessere, Università di Napoli Parthenope, 80133 Napoli, Italy; 4 Department of Movement Sciences and Wellbeing, University “Parthenope”, 80133 Naples, Italy

**Keywords:** cannabis, endocannabinoid system, reproduction, testis, spermatozoa, epigenetics

## Abstract

Cannabis is the most widely used illicit drug in Western counties and its abuse is particularly high in male adolescents and young adults. Its main psychotropic component, the cannabinoid delta-9-tetrahydrocannabinol (Δ^9^-THC), interferes in the endogenous endocannabinoid system. This signaling system is involved in the control of many biological activities, including the formation of high-quality male gametes. Direct adverse effects of Δ^9^-THC in male reproduction are well known in both animal models and humans. Nevertheless, the possibility of long-term effects due to epigenetic mechanisms has recently been reported. In this review, we summarize the main advances in the field suggesting the need to pay attention to the possible long-term epigenetic risks for the reproductive health of cannabis users and the health of their offspring.

## 1. Introduction

Spermatogenesis is a complex process leading to the production of male gametes, spermatozoa (SPZ). It occurs in testis and requires the coordination between mitosis, meiosis, and differentiation in order to maintain the continuum of sperm production. The process takes place from a pool of spermatogonial stem cells (SSCs) that self-renew and guarantees spermatogenesis progression through mitotic and meiotic divisions; at the end of meiosis, the newly formed haploid round spermatids undergo spermiogenesis, a differentiation process that transform them into SPZ [1,2,3]. However, mammalian SPZ undergo additional remodeling processes during the transit in the male and female reproductive tracts to gain all the features of fertilization-capable gametes [4,5].

Male reproductive health is notably under endocrine, paracrine, and autocrine control and is deeply affected by environmental cues and lifestyle [1,6,7,8,9,10,11]. In fact, environmental pollutants and endocrine disruptors, unbalanced diet, stressors, illicit drugs, abuse substances such as anabolic androgenic steroids or alcohol, smoking etc. exert adverse effects on reproduction with consequence on sperm production and quality [10,11,12,13,14,15,16,17,18,19,20].

**Figure 1:**
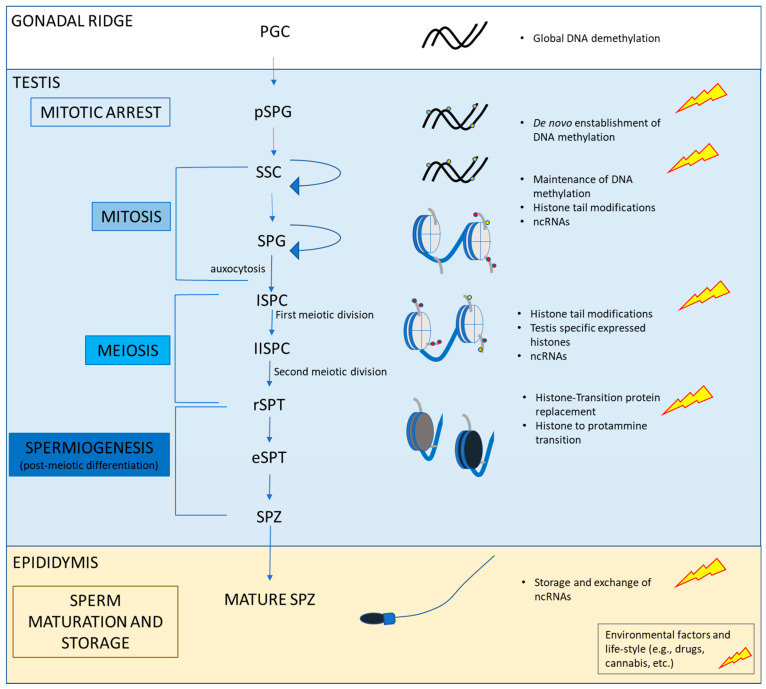
Main epigenenetic mechanisms occurring during the progression of spermatogenesis. During embryo development, Primordial Germ Cells (PGCs) migrate from the base of allantois to the gonadal ridges; in the developing testis they are committed to gonocytes (i.e., pro-spermatogonia) and undergo several rounds of proliferation before entering mitotic arrest. Then, male gonocytes migrate to the basal membrane of the seminiferous tubule and become spermatogonia. Starting from puberty, the potential spermatogonia stem cell pool can self-renew or differentiate into daughter cells, thus affecting the expansion of the mitotic pool and also committing spermatogonia toward the meiotic division. At the completion of meiotic division, the newly formed round spermatids undergo morpho-functional changes (spermiogenesis) and spermatozoa are released from testis. During the transit into the epididymis, spermatozoa acquire fertilization competence (i.e., motility) and are stored for ejaculation. The genome of PGCs is demethylated prenatally. The establishment of DNA methylation occurs in mitotically arrested spermatogonia in parallel to early redistribution of histone tail marks (further details in [25]). Then, DNA methylation status is maintained, and chromatin remodeling occurs via modification of histone tails and the expression of testis-specific histone variants. At spermiogenesis, histones are replaced by transition proteins and then transition proteins are replaced by protamines to get the hypercompact status of chromatin in SPZ. PGC: primordial germ cells; ISPC: primary spermatocytes; IISPC: secondary spermatocytes; SPG: spermatogonia; pSPG: gonocytes (i.e., pro-spermatogonia); eSPT: elongated spermatid; rSPT: round spermatid; SPT: spermatid; SPZ spermatozoa; SSC: spermatogonial stem cell pool. Histones are in light grey; transition proteins are in dark gray; protamines are in dark blue.

In the last years, particular attention has been paid to epigenetic changes [i.e., DNA methylation status at the CpG dinucleotide, histone tail changes such as methylation or acetylation, production of non-coding RNA (ncRNA)] that, as a result of lifestyle and environmental factors, modulate gene expression without any changes to the DNA sequence, thus making the conditions for possible disease load [13,21,22,23]. In this respect, an altered epigenetic signature in SPZ causes abnormalities in semen parameters and has negative consequences on fertilization, pregnancy rate, and early embryo development [24,25]. Nevertheless, the possible transmission of de-regulated epigenetic marks to the embryo via parental gametes has recently been reported [26,27]; consequently, the contribution of the male gamete to embryo has been revised with the introduction of paternal epigenetic inheritance [28,29]. Extensive epigenetic changes occur during spermatogenesis to produce high quality SPZ [24,30,31]. DNA methylation status is established in germ cells at an early stage and specific DNA methyltransferases guarantee its maintenance during gametogenesis progression. In parallel, chromatin remodeling via the chemical modification of histone tails and the expression of testis-specific histone variants (e.g., H3.3, H3t, TH2A/B etc.) both occur during the progression of spermatogenesis. This histone epigenetic signature is lost during spermiogenesis with the replacement of histone proteins by transition proteins (TNP1/TNP2), and subsequent replacement of transition proteins by protamines (PMR1/PMR2) [24,30,31]. Thus, for a long time, the epigenetic contribution of the SPZ to the zygote was considered to be very limited. However, the discovery of locus-specific retention of histones within the promoters of developmentally important genes demonstrated the presence of an epigenetic signature in SPZ suggesting a possible role in embryonic development [3,32,33]. Furthermore, RNA molecules, including ncRNA, that are expressed during spermatogenesis, can be stored in mature SPZ; in addition, during transit in the male reproductive tract, the communications between SPZ and somatic cells lead to the functional exchange of ncRNAs capable of modulating sperm functions and early embryo developmental stages when transferred to the zygote [25,30,34]. Hence, paternal experiences can be transmitted to the offspring through the male germline by epigenetic mechanisms. Figure 1 summarizes the main epigenetic changes during the spermatogenesis.

One of the main troubles in Western countries is the use of illicit drugs, such as cannabis and marijuana, for recreational use, especially among teenagers and young men [35,36]. These drugs interfere in the endogenous tone of key signalling molecules, collectively called endocannabinoids, that control the production of high-quality gametes in both sexes and pregnancy as an endpoint [37]. In this context, the ability of cannabis to heritably impact the sperm epigenome is poorly studied, but is highly relevant taking into consideration: (i) the higher recurrent cannabis consumption in boys and men than girls and women; (ii) the increasing use of cannabis as a therapeutic drug; (iii) the debate related to the increasing query of cannabis legalization for recreational use; (iv) the wrong perception of recreational cannabis as “safe” for health [35,36,38,39,40].

In this review, we summarize the main knowledge advances in the field providing evidence that long-term risks for the reproductive health of cannabis users and their offspring’s health deserve attention.

## 2. Cannabis: Introduction and Worldwide Use

Cannabis, known as an illicit drug, has been used for many decades, for recreational, medical, and industrial use [41,42]. Moreover, medicinal cannabis is a therapy that has garnered much national attention in recent years, due to controversies about legal, ethical, and social implications associated with its use [43,44].

Cannabinoids are natural substances, derived from Indian hemp (*Cannabis sativa*), as well as having synthetic analogues (similar substances produced synthetically). *Cannabis sativa* is an annual dioecious plant, which shares its origins with the beginning of the first agricultural human societies in Asia [45]. In the course of time, different parts of the plant have been used for medical and recreational purposes, for example, extraction of healing oils from seed, or the use of inflorescences for their psychoactive effects. The most active substance among these compounds is delta-9-tetrahydrocannabinol (Δ^9^-THC), which is the main psychoactive ingredient in cannabis [46]. Marijuana is a substance derived from *Cannabis sativa* and *Cannabis indica* plants that contains Δ^9^-THC and other chemicals related to Δ^9^-THC exhibiting psychotropic and non-psychotropic effects. There are several pharmaceutical cannabis products available, but herbal preparations are the most widely used [47]. However, the properties of several phytochemicals in cannabis and marijuana are still poorly known [48,49]. Cannabis has been used for centuries for beneficial purposes. This plant has recently seen a resurgence of interest due to its multi-purpose applications; it is indeed a treasure trove of phytochemicals and a rich source of both cellulosic and woody fibers [47,48,49,50,51,52].

There are more than 460 compounds recognized in cannabis, and more than 140 are phytocannabinoids, which are a unique group of C_21_ terpenophenolic natural products [53]. Currently, there are thousands of cannabis types on the market with different compositions of phytocannabinoids; they are classified and marketed based on the total amount of Δ^9^-THC and cannabidiol (CBD) [54]. The main phytocannabinoids are listed in Table 1.

CBN was the first pure phytocannabinoid isolated from cannabis in 1899; it is mildly psychoactive and has several pharmacological activities [55].

CBD is the second most abundant cannabinoid present in *Cannabis sativa*. For its safe therapeutic profile and its deficiency of psychoactivity, CBD is one of the most interesting compounds, with a lot of reported pharmacological effects in several pathological situations from inflammatory and neurodegenerative diseases to epilepsy, autoimmune disorders such as multiple sclerosis, arthritis, schizophrenia, depression, cancer, and many others. Moreover, there is evidence to support the use of endogenously produced cannabinoids as well as phytocannabinoids broadly that exhibit anti-inflammatory actions in association with anti-inflammatory drugs [56,57,58,59]. In 2018 the United States Food and Drug Administration approved a CBD oral solution under the trade name Epidiolex^®^ for the treatment of Lennox–Gastaut syndrome and Dravet syndrome, two rare and severe forms of epilepsy, in paediatric patients. Furthermore, dose–response effects have also been noted for testicular cancer: cannabis exposure intensely accelerates the preclinical oncogenic phase from several decades to less than about 14 years [60,61].

Drug use data for cigarettes, alcohol use, analgesics, cannabis, and cocaine were taken from the National Survey of Drug Use and Health, a nationally representative study conducted annually by the Substance Abuse and Mental Health Services Administration (SAMHSA) with a 74.1% response rate. According to the United Nations Office on Drugs and Crime, World Drug Report 2021, cannabis was used by an estimated 200.4 million people (95% CI 141.4 to 256.4 million) worldwide in 2019, approximately 4.0 percent (95% CI 2.8 to 5.1 percent) of the global population aged 15 to 64 years [35,44].

Cannabis for smoking represents the most common illegal drug in the world. There are an estimated 140–150 million cannabis users worldwide as compared to rough estimates of 14–15 million for cocaine and 13–14 million for opium, heroin, and other opioid drugs [62].

Some studies show large increases in cannabis use prevalence of 25% to 82% in age strata 15–24 years and 55–64 years, respectively, and a modest to intense increase across 2010–2019, thus resulting in the most widely used illicit drug among adolescents and young adults. High-risk daily use also increased in many nations and by 600%, 48% and 45% in Portugal, Spain, and France, respectively. The concentration of Δ^9^-THC in cannabis herb and resin increased 54% and 217%, and in nations such as France, Sweden, and Luxembourg, Δ^9^-THC concentrations in cannabis resin rose 2.3–2.5% annually [63].

The main synthetic cannabinoid intoxication effects are summarized in Table 2.

## 3. The Endogenous Endocannabinoid System

(Phyto)Cannabinoids apply their effects by interacting with specific cannabinoid receptors. Classically, there are two main cannabinoid receptors in the body: CB1 (first discovered in the central nervous system, CNS) and CB2 (first discovered in peripheral tissues). These G protein-coupled receptors can be activated by endogenous ligands (endocannabinoids) such as arachidonoylethanolamide (AEA), known as anandamide and 2-arachinodyl-glycerol (2-AG), the first endocannabinoids discovered in biological systems. Endocannabinoids are synthesized and degraded by separate pathways. Essentially, endocannabinoids are lipid molecules mainly derived from membrane phospholipids. Cannabinoid receptors, together with hydrolyzing and biosynthetic enzymes and transporters, constitute the classical endogenous endocannabinoid system [75,76,77,78]. In recent years, the spectrum of cannabinoid signaling enlarged with the discovery of additional cannabinoid receptors such as GPR55, non-canonical cannabinoid receptors [e.g., the cationic channel type 1 vanilloid receptor (TRPV1), the nuclear peroxisome proliferator-activated receptor γ (PPARγ), or the γ-aminobutyric acid (GABA) receptor A subtypes], and lastly endocannabinoid-like substances [75,76,77,78].

In the CNS, the endocannabinoid system is involved in the control of synaptic transmission and plasticity, neurogenesis, learning and memory, wake–sleep cycle, thermogenesis, pain, mood, etc. but also neuroendocrine functions including stress response, food intake, and reproduction [37,79,80,81]. In the CNS endocannabinoids are not stored in vesicles like classical neurotransmitters but are rapidly synthesized by neurons in response to the entry of calcium ions or the activation of metabotropic receptors (e.g., acetylcholine and glutamate receptors). Endocannabinoids can function as retrograde messengers at the synaptic level: they are released by postsynaptic neurons and move retrogradely by activating the CB1 receptors located on the postsynaptic neuron, thus reducing the release of neurotransmitters. With this mechanism, exogenous cannabinoids such as Δ^9^-THC can alter memory, cognitive functions, psychomotor performance, and pain perception. The therapeutic potential of cannabis and its psychoactive effects have been known for many years [82,83]. Therefore, the effects of cannabinoids depend on the brain area involved.

After oral administration, Δ^9^-THC is almost completely absorbed but undergoes an important hepatic first-pass metabolism. Its metabolites are slowly eliminated over days to weeks in the faeces and urine. Marijuana is a psychoactive agent used in medicine to stimulate appetite and as an antiemetic even if the mechanisms underlying these effects have not been defined as more effective drugs are available and rarely used in combination with other antiemetics. A recent study indicating additional mechanisms by which endocannabinoid signalling controls Dopamine (DA) function (CB2 receptor regulation of DA function) suggests these two systems are even more unified than previously thought [84]. Experimental studies on the behavioral and biochemical properties of Δ^9^-THC and its analogues confirm the status of cannabis as an addictive substance not unlike that produced by the so-called hard drugs, such as heroin, which shares the stimulation of the mu opioid receptor with cannabis [85].

Lastly, the widespread distribution of the endocannabinoid system in biological tissues, in particular in the brain areas linked to conditioning processes such as alcohol addiction or drug-seeking behavior, has encouraged research into the epigenetic modulation of the system revealing how this is highly sensitive to environmental epigenetic cues [18]. In this respect, the understanding of how gene expression is spatio-temporally regulated by the epigenome is relevant to study the micro-environment and dynamic processes that regulate cell identity, state, and fate in correlation to lifestyle and environmental factors. Recent technological analyses such as single-cell RNA analysis/sequencing and spatial transcriptomics have been developed and proved very useful to generate data on the expressed genes in a single cell and within tissues for spatially deciphering cellular composition, heterogeneity, and cell–cell communications [86]. This approach complements with epigenomics to profile long-term epigenetic changes in histone tails and DNA marking active or silent promoters, putative enhancers, DNA accessibility to transcription and binding of transcription factors, promoter–enhancer interactions, and three dimensional genome organization in individual cells [87,88]. At present, single cell RNA sequencing offered unprecedented tools to analyze the transcriptome after the modulation of the endocannabinoid system in health and disease. The effects of Δ^9^-THC on gene expression have been reported in peripheral blood mononuclear cells [89]; the effects of AEA and the precise cells and molecular signatures involved in the Δ^9^-THC-mediated induction of apoptosis in Acute Respiratory Distress Syndrome have been observed [90,91]; CBD dependent modulation of neuroinflammation and intestinal inflammation in autoimmune encephalomyelitis [92], and the transcriptome in hippocampal and cortical cells following the inhibition of 2-AG metabolism [93] have been recently reported.

## 4. Preconception Exposure to Cannabis: Epigenetic Effects in SPZ

The endocannabinoid system affects reproduction at multiple levels in both sexes effecting the production of high-quality gametes and pregnancy outcomes [37,75,79,80,81,94]. Centrally it modulates the release of Gonadotropin Releasing Hormone (GnRH), the decapeptide responsible for gonadotropin discharge and gonadal sex steroid production. In the gonads, it step-by step mediates spermatogenesis progression, Leydig and Sertoli cell functions in males, and folliculogenesis, oocyte maturation, and ovulation in females [37,75,79,80,81,94]. In reproductive tracts, endocannabinoid signaling controls sperm maturation, acrosome reaction, fertilizing ability, early embryo development, implantation, embryo growth, and delivery [37,75,79,80,81,94]. In this respect the modulation of endocannabinoid tone by hydrolyzing enzymes is the most critical step in endocannabinoid signaling.

Cannabis use interferes in this endogenous system, reducing reproductive ability and pregnancy as an endpoint. For a long time, the effects of marijuana smoking and Δ^9^-THC have been studied in humans and animal models. These studies discovered a plethora of deleterious effects, such as the impairment of the hormonal milieu controlling reproduction in both sexes, reduced SPZ quality, impaired ovulation, disruption of menstrual cycle, intrauterine fetal growth restriction, increased incidence of preterm birth etc. [37,75], but significant bias in humans occurred.

Recently, the epigenetics of the endocannabinoid system by lifestyle, phytocannabinoids, or drugs (i.e., specific agonists and antagonists) has been reviewed [18], revealing that the system is both an epigenetic target and a modulator of epigenetic machinery and gene expression in tissues with the possibility of (endo/phyto)cannabinoid-dependent trans-generational epigenetic inheritance in the offspring [18]. Hence, attention towards the male gamete that not only contributes to fertilization with a haploid nucleus, but also provides an epigenetic signature directly linked to paternal experiences, highlights potential risks induced by recreational cannabinoid exposure.

Δ^9^-THC exposure during pregnancy has several adverse outcomes, such as low body weight at birth [95], but also developmental and behavioral troubles particularly neurodevelopmental, musculoskeletal, and cardiovascular defects, or deregulation of innate and adaptive immune system, or congenital/teratogenic defects in children [96,97,98,99,100,101,102]; however, the ability of cannabis to heritably impact the sperm epigenome is poorly studied, but highly relevant.

In the last 5 years, few studies analyzed the cannabis-related epigenetic changes in human SPZ revealing the association between cannabis use, sperm concentrations, and widespread changes in sperm DNA methylation [103,104]. However, the follow up on these epigenetic changes in the offspring is quite unrealistic in humans, and thus the use of experimental animal models, particularly rodents, has been very useful.

In this respect, data in rodents revealed that preconception paternal Δ^9^-THC exposure elicits deficits in cholinergic synaptic function [105], abnormal loco-motor activity, impaired cognitive functions, or long-lasting neurobehavioral effects [106,107,108] in their offspring; similarly, Δ^9^-THC exposure in adolescent rats caused cross-generational effects in DNA methylation status in the nucleus accumbens (NAc) [109]. In the last years, the potential epigenetic role of the father in the transmission of behavioral troubles such as autism or autism-like phenotypes to the offspring have been investigated in animal models and recently in humans [103,104], revealing as epigenetic target the paternally imprinted gene *Discs-Large Associated Protein 2* (*DLGAP2).* This gene encodes a membrane protein located on postsynaptic neurons involved in neuronal signaling and synapse organization [110], and is known as an autism/autism spectrum disorder (ASD) and schizophrenia candidate gene [110,111]. Interestingly, results showed that *DLGAP2* was hypomethylated at nine validated CpG sites within intron 7 in the SPZ from marijuana smokers and Δ^9^-THC exposed rodents. Furthermore, DNA methylation changes in *DLGAP2* were also confirmed in the NAc of the offspring of, and related to the possible development of autism-like phenotype in rats born from, fathers exposed to Δ^9^-THC prior to conception [104]. In addition, Garrido et al. [112] carried out a genome-wide analysis for differential DNA methylation regions in sperm samples obtained from fathers that have children with or without autism (N = 13/group) revealing a total of 805 DNA methylation regions in known ASD genes, as well as other neurobiology related target genes [112]. Interestingly, 612 of these DNA methylated regions parallel the 127 sites that differ significantly between users and controls after cannabis abstinence [113]. The list of common genes also includes *DLGAP2*, the gene hypomethylated in the sperm of cannabis users and related to paternal trans-generational inheritance of autism in rodents [103,104].

The effects of Δ^9^-THC administration by oral gavage or intraperitoneal injection on sperm DNA methylation in seven genes notably involved in neurodevelopment and implicated in autism and ADS autism-like phenotypes (i.e., *Dlg4*, *Grid1*, *Lrrtm4*, *Nrxn1*, *Nrxn3*, *Shank1*, and *Syt3*) were recently compared to each other and to nicotine exposure in rats [114]. Administration route dependent effects, and different outcomes on DNA methylation levels in the same target genes, were observed. Therefore, neurodevelopmental genes were shown to be highly sensitive to epigenetic modulation, but the extent of epigenetic changes depends on both drug type and administration route [114].

An additional question, with important public health implications, is the possible reversibility of epigenetic changes in the semen through a “washout” period. Among epigenetic changes, DNA methylation status is particularly interesting in that specific DNA maintenance methyltransferases are responsible for the high-fidelity maintenance of DNA methylation during the mitotic events that characterize the self-renewal of intratesticular stem cells (i.e., spermatogonia). Conversely, epigenetic changes occurring during the progression of spermatogenesis, particularly at spermiogenesis, may result in being transient and not fixed in all gametes due to the continuous production/release/reabsorption of SPZ [24,30,31].

Two recent studies by Schrott and co-workers [113,115] investigated this interesting issue. The authors used rat model to expose fathers to cannabis at different conditions (early and later exposure in combination with a 56-day long “washout” period, to complete just one spermatogenetic cycle); then, they analyzed the DNA methylation profile in the semen of the exposed fathers (F0) and the paternal derived epigenetic changes in offspring semen and tissues [115]. Prior to mating, male rats were exposed early to cannabis extract for 28 days and then received a “washout” period; conversely, in later exposure, rats first received the “washout” period and then were exposed to cannabis extract for 28 days. Most DNA methylation changes occurred in the same direction in early and later exposed animals. However, the timing of cannabis exposure affected the magnitude of epigenetic changes since DNA methylation changes diminished following the 56-day long abstinence. Thus, it is plausible that cannabis abstinence for one spermatogenetic cycle may be useful to attenuate the effects on sperm DNA methylation profile. However, some DNA methylation changes specifically occurred after early exposure only and persisted after the cannabis wash-out period, probably through epigenetic changes in the pool of staminal spermatogonia. The phenotype of rats born to cannabis extract exposed fathers was characterized by significant cardiomegaly. Whole genome bisulfite sequencing (WGBS) revealed 3321 CpG islands with altered methylation levels in the semen of F0 exposed rats; the methylation status of selected genes (i.e., *2-Phosphoxylose Phosphatase 1* (*Pxylp1*), *Metastasis Suppressor 1-Like Protein* (*Mtss1l*)) was validated via bisulfite pyrosequencing. Interestingly, as in the sperm of exposed parents, significant DNA methylation changes at the *Pxylp1* gene were detectable in the SPZ of their offspring; in parallel, significant DNA methylation changes at the *Mtss1l* gene were detectable in the hippocampus and NAc of cannabis exposed offspring, with a sex-specific relationship between DNA methylation and gene expression in the NAc [115].

In the second study, the same group demonstrated that 11 weeks of cannabis abstinence, a period that corresponds to the duration of one spermatogenic cycle in humans, diminished cannabis-associated changes in DNA methylation in human sperm from 163 to 127 CpG sites [113]. In this study, a total of 163 CpG sites in genes involved in developmental processes such as cardio-genesis and neurodevelopment, with significantly different DNA methylation status, were first identified by WGBS in the sperm of controls, and at least once weekly, over cannabis users (N = 24 and N = 18 respectively, both 18–40 years old). Then, following a 77-day long period of cannabis abstinence, the methylation changes at the selected developmental genes were analyzed in the SPZ epigenome revealing significantly reduced methylation levels vs. those detected in cannabis users; however, 127 CpG sites still significantly differed between users and controls after abstinence, suggesting that long-term and not transient effects occurred in germ cells. Despite being limited to a small number of subjects, this preliminary study in humans provides many clinical implications such as the need to stop using cannabis prior to conception to preserve gamete epigenome or the possibility to perform sperm methylome analysis to assess the risk of transmission to the offspring of deregulated epigenetic marks.

Lastly, the direct effects of cannabis exposure on DNA methylation status at specific target genes was tested in human spermatogenesis in vitro demonstrating altered DNA methylation at the maternally imprinted genes *SGCE* and *GRB10* in spermatogonial stem cells and spermatid-like cells, respectively; differential methylation of *PEG3* in spermatid-like cells only; and altered DNA methylation levels of *HCN1* and NR4A2—two ASD-related genes—in spermatogonial stem cells only [116].

**Figure 2:**
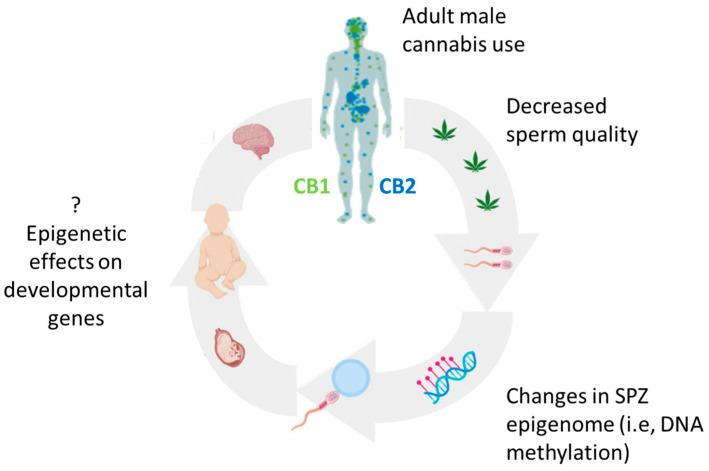
Distribution of cannabinoid CB1 (mainly localized in the brain—hippocampus, cerebellum and cerebrum, green dots) and CB2 receptors (mainly situated in the periphery—spleen, tonsillar, and immune cells, blue dots). Summary of cannabis exposure and probable consequences.

Taken together, the possible risk related to the use of Δ^9^-THC containing products or cannabis extracts during the preconception phase may have tangible consequences on reproduction and offspring health (Figure 2).

## 5. Conclusions

Male teenagers and young men are the main users of cannabis in Western countries. This substance interferes in the endogenous tone of endocannabinoids, one of the major controllers of reproduction and fertility in both sexes. Upcoming evidence from humans and animal models revealed that cannabis and its main psychoactive compound, Δ^9^-THC, affect the sperm epigenome, particularly at the DNA methylation level, with possible effects on developmental genes such as *DLGAP2* and consequences on the heath of offspring. In particular, studies on epigenome screening by both WGBS and reduced representation bisulphite sequencing carried out in human and rodent SPZ revealed conserved epigenetic targets following marijuana/Δ^9^-THC exposure; furthermore, possible partial rescue of the physiological DNA methylation levels at selected gene loci occurred following drug abstinence. Research in the field is still limited but upcoming data deserve attention for the evaluation of health risk. In recent years, the use of cannabis as a therapeutic drug or for recreational purposes has increased. Similarly, the increasing query of cannabis legalization for recreational use is leading to the wrong perception of recreational cannabis as “safe” for health. Further studies are surely needed in the field, but reproductive health risk must be taken into consideration especially at pre-conception.

## Figures and Tables

**Table 1:** The most common phytocannabinoids.

Phytocannabinoid
Δ^9^-trans-tetrahydrocannabinol (∆^9^-THC)
Δ^8^-tetrahydrocannabinol (∆^8^-THC)
cannabigerol (CBG)
cannabichromene (CBC)
cannabidiol (CBD)
cannabinodiol (CBND)
cannabielsoin (CBE)
cannabicyclol (CBL)
cannabinol (CBN)
cannabitriol (CBT)
other cannabinoids

**Table 2:** Reported effects and intoxication with synthetic cannabinoids.

System	Cannabinoid Drugs and Their Adverse Effects	References
Cardiac	Tachycardia, supraventricular tachycardia, ventricular fibrillation, myocardial infarction, coronary arterial thrombosis	[64,65,66]
Neurological	Dizziness, drowsiness, tremor, altered mental status, seizure, acute ischemic infarction, memory deficit	[10,50,54,56,57,58]
Psychiatric	Agitation, anxiety, paranoia, psychosis, suicidal ideation, delirium, dissociation, depersonalization, hallucinations, disorganized behaviour	[42,46,56,58]
Haematological	Immune thrombocytopenia, intracranial haemorrhage, coagulopathy	[67,68,69]
Others	Nausea, vomiting, hyperthermia, acute kidney injury, testosterone synthesis inhibition, reduced spermatogenesis, cancer	[37,70,71,72,73,74]
